# Editorial: Autism spectrum disorder: exploring the speech and language continuum

**DOI:** 10.3389/fnhum.2026.1972957

**Published:** 2026-09-29

**Authors:** Maria Mody, Margot J. Taylor, Christopher J. McDougle

**Affiliations:** 1 MGH Lurie Center for Autism, Lexington, MA, United States; 2 MGH Martinos Center for Biomedical Imaging, Charlestown, MA, United States; 3 Harvard Medical School, Boston, MA, United States; 4 Diagnostic and Interventional Radiology, Hospital for Sick Children, Toronto, ON, Canada

**Keywords:** approaches, autism, language, metrics, speech

About one-third of individuals on the autism spectrum are considered minimally verbal. Among the remaining two-thirds, linguistic ability can vary widely in form, content and/or use, consistent with a model put forth by Bloom and Lahey (1978). Disruptions in one or more of these domains can interact to give rise to spoken language phenotypes varying in profile and severity. In fact, individuals on the spectrum have been described as non-speaking, minimally verbal (mvASD), or speaking very well but having difficulty with the social rules of language. Adding to the complexity are comorbid conditions like Apraxia of Speech, Intellectual Disability, and ADHD that could contribute to the atypical speech and language continuum in ASD. A focus on spoken language has further muddied the waters regarding the field's understanding of the ability to communicate, especially in individuals with mvASD, some of whom appear to be able to express themselves independently via keyboards or letterboards (NIDCD/NIH workshop panel, Jan 24–25, 2023).

In this Research Topic, the collection of papers presents different approaches to study speech and language across the spectrum through the lens of basic and intervention findings. What appears to emerge is the potential for language in ASD to lend itself to analyses revealing nuanced impairments at basic syllable and higher discourse levels, reflecting the heterogeneity and complexity of the disorder. The studies propose metrics to detect and track autistic behaviors toward phenotyping of communication patterns. With the growing call for the recognition of Profound Autism as a distinct diagnosis, underrepresented in ASD research, finding ways not just to assess and offer interventions for these individuals but to test the robustness of our metrics early on in development as predictors of long-term outcomes has gained momentum.

In a recent study, Pecukonis et al. found that fine motor skills predicted expressive but not receptive language abilities in adults with mvASD, in support of a role for the motor system in verbal ability, and its impact on social emotional skills across the spectrum. Perhaps then, a discrepancy between receptive and expressive language in ASD might have its origins in poor motor skills, evident early on in autistic children. Given the contributions of both social and motor skills to language development, there is a clear need for empirical and operationalizable measures of these abilities that can be used in children and adults to assess, track, compare and interpret changes across the spectrum.

It is well known that speech and language findings vary with the subgroup under study. Kato and Hanawa (a) and Kato and Hanawa (b) examined linguistic discourse abilities in verbal individuals with ASD, focusing on lexico-grammatical profiles to distinguish between ASD and non-ASD. Among the markers driving the distinction between the groups was a reduced use of inferential structures and syntactic patterns indicating deficits in joint attention with conversational partners, challenges in expressing agency and a preference for repetitive syntactic structures. The approach provides a linguistically grounded perspective of ASD cognition for use in intervention, while providing insights into the cognitive mechanisms underlying language use in ASD.

In contrast, acoustic and surface electromyography (EMG) investigations of speech and language in mvASD (Protyasha et al.; Quatieri et al.) focused on syllable and single word production tasks constrained by the limited speech in this population. At the core of the approach used in these two papers is the concept of motor coordination across the three speech subsystems, respiratory, laryngeal and articulatory systems, and the coupling among them as a measure of complexity of speech gestures. That the articulatory base features were most consistent in correlating with expressive vocabulary across tasks and participants [neurotypicals (NT) and mvASD] may further support a role for fine motor skills in the speech challenges of individuals with mvASD. The EMG data demonstrated stronger correlation between sensor signals from muscles on the face and neck on all tasks potentially related to tightly constrained speech gestures with fewer degrees of freedom in the mvASD group reflecting reduced complexity. These studies provide novel metrics for evaluating speech production abilities and have the potential to be applied beyond syllables and words to phrase and sentence level analysis, serving as a bridge to suprasegmental features like intonation contours. An interesting question is whether the flat prosody frequently seen in individuals with ASD may be related to disruptions in auditory feedback, interacting with reduced vocal pitch control. In a separate but potentially related series of questions, Trice and Qi examine how the autistic child encodes and retrieves novel words and what drives individual differences. They found evidence not just for pragmatic inferences but direct mapping in facilitating acquisition of word meaning in ASD. Findings across all these studies appear to support the use of top-down and bottom-up strategies in ASD as a function of cognitive linguistic profiles across the spectrum.

Given the hallmark difficulties in speech and language in individuals with ASD, there has been a growing interest in the identification of neurobiological markers of these challenges. White matter findings by Slušná et al. and Yi et al. in this Research Topic series, revealed significant microstructural differences between children with mvASD and typically developing (TD) control groups, linking myelin abnormalities to ASD and Diffusion Tensor Imaging (DTI) metrics to ASD symptom severity. However, while there was clear evidence for white matter integrity in support of language abilities in young children with ASD, the microstructural differences were not restricted to the language-related tracts but extended to cerebrospinal fluid (CSF) in children with non-verbal ASD. This highlights the challenges of interpreting neurobiological differences in those with ASD who are non-speaking or minimally verbal. The observations further emphasize the importance of methodological rigor, longitudinal studies, and pre-post intervention differences to meaningfully relate white matter alterations as a biomarker for speech and language in ASD. Pre-post differences in behavioral performance can also serve as a measure of efficacy of treatment. Hu et al. found that PROMPT-based training, implemented over seven steps by trained speech therapists, using a strict hierarchical approach and focused on speech motor control, was associated with better outcomes than parent implemented home-based language-cognitive training in children with ASD and speech language delay. These findings provide indirect support for the EMG and acoustic findings mentioned earlier, that implicate motor speech coordination difficulties in adults with mvASD. Importantly, though, the baseline communication abilities of children with ASD with speech and language impairments must be taken into consideration in designing and assigning intervention groups. In a study of toddlers' response to caregiver-mediated interventions, Brian et al. found that toddlers' functional communication inversely predicted their caregivers' mastery of intervention strategies. Conversely, higher baseline receptive communication skills predicted better gains in the toddlers' responsivity. These results, when examined in the context of findings by Hu et al., highlight the importance of establishing the baseline language skills of children with ASD participating in caregiver mediated interventions to help match them with personalized intervention approaches.

Despite the complexity of the speech and language challenges in ASD, the responsibility lies with the clinical and research community alike to ensure that the voices of those on the spectrum are heard. This may include making necessary adaptations such as the inclusion of visual supports to assist the autistic individual with answering interview questions, as in the adaptation of the Autism Voices framework by Beauchamp et al. Additionally, as Żuromski and Pacolek-Żuromska remind us, the mind is a product of the interaction with culturally structured environments. As such, the autistic mind would also reflect the challenges that individuals with ASD may have in interpreting and engaging with the social world, leading to a myriad of downstream consequences.

In summary, the collection of papers in this Research Topic presents new approaches to investigating communication in ASD through the lens of basic, intervention, and neurobiological findings across the spectrum. The insights gained from these approaches could help guide future efforts to capture real world everyday social interactions through naturalistic observations coded for communication intent, a key piece in the development of personalized devices to enhance communication in individuals on the spectrum.
